# Implications of Patient Portal Transparency in Oncology: Qualitative Interview Study on the Experiences of Patients, Oncologists, and Medical Informaticists

**DOI:** 10.2196/cancer.8993

**Published:** 2018-03-26

**Authors:** Jordan M Alpert, Bonny B Morris, Maria D Thomson, Khalid Matin, Richard F Brown

**Affiliations:** ^1^ Department of Advertising University of Florida Gainesville, FL United States; ^2^ Department of Health Behavior and Policy Virginia Commonwealth University School of Medicine Richmond, VA United States; ^3^ Division of Hematology and Oncology, Department of Internal Medicine Virginia Commonwealth University School of Medicine Richmond, VA United States

**Keywords:** qualitative research, electronic health record, patient portals, physician patient relationship, health communication

## Abstract

**Background:**

Providing patients with unrestricted access to their electronic medical records through patient portals has impacted patient-provider communication and patients’ personal health knowledge. However, little is known about how patient portals are used in oncology.

**Objective:**

The aim of this study was to understand attitudes of the portal’s adoption for oncology and to identify the advantages and disadvantages of using the portal to communicate and view medical information.

**Methods:**

In-depth semistructured interviews were conducted with 60 participants: 35 patients, 13 oncologists, and 12 medical informaticists. Interviews were recorded, transcribed, and thematically analyzed to identify critical incidents and general attitudes encountered by participants.

**Results:**

Two primary themes were discovered: (1) implementation practices influence attitudes, in which the decision-making and execution process of introducing portals throughout the hospital did not include the input of oncologists. Lack of oncologists’ involvement led to a lack of knowledge about portal functionality, such as not knowing the time period when test results would be disclosed to patients; (2) perceptions of portals as communication tools varies by user type, meaning that each participant group (patients, oncologists, and medical informaticists) had varied opinions about how the portal should be used to transmit and receive information. Oncologists and medical informaticists had difficulty understanding one another’s culture and communication processes in their fields, while patients had preferences for how they would like to receive communication, but it largely depended upon the type of test being disclosed.

**Conclusions:**

The majority of patients (54%, 19/35) who participated in this study viewed lab results or scan reports via the portal before being contacted by a clinician. Most were relatively comfortable with this manner of disclosure but still preferred face-to-face or telephone communication. Findings from this study indicate that portal education is needed for both patients and oncologists, especially when portals are implemented across entire health systems since highly specialized areas of medicine may have unique needs and uses. Patient portals in oncology can potentially alter the way diagnoses are delivered and how patients and oncologists communicate. Therefore, communication about the portal should be established during initial consultations so patients can decide whether they want to be informed in such a manner.

## Introduction

The uptake of patient portals by health systems is growing across the United States due to recommendations by the Institute of Medicine [1] and positive patient responses from accessing their electronic medical records through portals [2]. However, some providers remain skeptical about patient’s unrestricted access to such medical information and have expressed concerns about the legal and ethical ramifications of patient portal usage [3]. In addition, studies analyzing physician’s attitudes toward patients viewing medical information through portals found that there was concern that patients who were largely untrained and unprepared to view abnormal medical results [4,5] may be confused [5] and highly anxious [6] about their health condition.

Patient portals are relatively new applications, yet they are increasingly being offered to patients despite a limited number of studies that provide detail on successful implementation practices [7]. Moreover, the majority of research exploring experiences of patients using portals has focused on the primary care setting [8-13]. Primary care patients predominantly use patient portals to view doctor’s notes, understand their condition, and to check for errors in their record [14]. Primary care patients have reported high levels of satisfaction when viewing lab results online [9]. However, patients favored office visits over Web portals for learning about abnormal cancer tests [15]. Face-to-face disclosures of cancer diagnoses and prognoses allow patients to express concerns, resulting in lower anxiety and depression [16-19].

The use of patient portals is growing in the oncology setting [20]. Cancer patients desire test results in the most rapid manner possible [21] and also rate the importance of electronic access to retrieve their medical records higher than patients without cancer [22]. However, in contrast to typical primary care patients, cancer patients who view results on patient portals may potentially discover that their cancer has grown or metastasized. This is particularly concerning as many cancer patients do not fully understand their prognosis [23]. Moreover, patients are viewing this information at a time of heightened emotional distress, characterized by fear and uncertainty, exacerbated by the complexity of the information [24]. Thus, distress levels of cancer patients may be compounded by unfettered access to their medical record through patient portals.

To illuminate our knowledge of the potential advantages and disadvantages of portal usage by cancer patients, we obtained the perspectives of key stakeholders—patients, oncologists, and medical informaticists. Since little is known about how cancer patients and other stakeholders utilize the portal, the goals of this study were (1) to understand attitudes about the adoption of the patient portal for oncology and (2) to explore the potential implications of patient portal usage as a method of communication in oncology.

## Methods

### Study Setting

This study took place at a National Cancer Institute designated cancer center in central Virginia. In June 2015, patient portals began displaying pathology results, doctor’s notes, and after a 4-day delay, radiology reports. Over 70,000 patients across the entire health system are connected to the patient portal. Recruitment for this study occurred between May and September 2016. This study was approved by the local Institutional Review Board of Virginia Commonwealth University.

### Participants

#### Oncologists

Members of the research team presented an overview of the study at hematology, radiation and surgical oncology service meetings. Out of the 46 oncologists present during the meetings, all agreed to be contacted in the future for potential study participation. Almost half (22/46, 48%) of the oncologists were randomly selected and then recruited through an email invitation to participate in the study. Informed consent was reviewed with willing participants, and written consent was obtained before the face-to-face interview.

#### Medical Informaticists

In total, 5 medical informaticists were recruited from the local health system. Among the 5 informaticists, 3 were members of the original patient portal committee that recommended its adoption, and 2 were involved in decisions regarding portal usage at the same institution. All 5 informaticists were contacted by email to participate in a face-to-face interview and written consent was obtained from them. To gain a broader perspective of opinions about portal transparency beyond the local health system, we sought viewpoints from external informaticists to either validate or provide alternative claims using purposeful snowball sampling [25]. Local medical informaticists referred 7 medical informaticists and chief medical information officers (CMIOs) at 5 health systems across the country utilizing similar information technology systems. An email invitation was sent that mentioned the referring medical informaticist and a description of the study. Informed consent was reviewed with potential participants who responded to the email, and verbal consent was given over the phone before interviews commenced.

#### Patients

Research staff identified potential patient participants using clinic schedules. Patients were eligible if they were (1) registered and enrolled in the portal, (2) fluent in English, (3) able to provide informed consent, (4) at least 21 years of age, and (5) had attended an appointment with a participating oncologist within the previous 4 weeks from patient identification.

From clinic schedules of oncologists, 72 eligible patients were randomly selected. In addition, purposive sampling [26] was used to recruit 6 patients, who were referred by participating oncologists since they reported a negative experience with the portal. All 78 patients were mailed a letter explaining the study’s purpose, with a form to opt-out of further contact coupled with a self-addressed stamped envelope. If no opt-out form was received until 2 weeks of the letter being mailed, a member of the research team contacted the patient by telephone to discuss study requirements, obtain verbal assent to participate, and to set up a time for the phone interview. Patients received a US $25 gift card as appreciation for their participation.

### Procedure

This study employed qualitative in-depth respondent interviews [27,28]. A semistructured interview guide was developed using the critical incident technique (CIT) [29], which is a qualitative research approach to collect information about significant incidents related to an event [30]. CIT has been used to analyze quality of care [31] and the applications of health care services [32]. CIT was employed by asking open-ended questions to elicit specific, in-depth details about respondent’s encounters with patient portals. The semistructured interview guides were modified to enable use in each stakeholder group (oncologists, medical informaticists, and patients) and were designed to prompt their personal and professional experiences. For instance, patients were asked neutral questions about their experiences of using the portal to view their medical information, while oncologists were asked about their experiences with respect to patients using the portal. Similarly, all 3 groups were asked variations of the question, “how has viewing/inputting health information on the portal changed the way you interact with oncologists/patients?” The questionnaire was designed to have participants describe a situation, explain its significance, and specify the eventual outcome [33]. Interviews were audio-recorded, and ATLAS.ti (version 7.5, Scientific Software Development GmbH [34]) was used to manage the verbatim transcripts and coding process.

### Data Analysis

The research team analyzed the transcripts verbatim using an iterative, thematic text analysis approach to best describe different stakeholder perspectives [35]. In the beginning, 2 members of the research team individually read 9 transcripts, 3 from each group, and began to develop preliminary codes [28]. They met weekly to compare coded transcripts, discuss discrepancies, and define codes that were compiled into a shared code book used by each coder on subsequent transcripts [36]. Subsequently, the entire research team gathered to synthesize, describe, and systematically group codes into larger-order thematic classifications. As part of this process, themes were compared across stakeholder groups to identify similarities and dissimilarities in experiences and attitudes. The authors confirm that all participant identifiers have been removed or disguised, so the participants described are not identifiable and cannot be identified through the details of their quotes.

## Results

### Demographics

Of the 60 participants enrolled, the enrollment rate was 59% (13/22) for oncologists, 92% (12/13) for medical informaticists, and 45% (35/78) for patients. Table 1 contains detailed recruitment information. Of the 13 oncologists enrolled, 8 specialized in hematology/oncology, 4 in radiation oncology, and 1 in surgical oncology. The average age of the oncologists was 47 years, 54% (7/13) were women, and 77% (10/13) were white. Oncologists had an average clinical practice of 14 years, ranging from 3 to 33 years.

Half of the 12 medical informaticists were the CMIO at their institution, and the rest were clinicians trained as informaticists. The average age was 54 years with 24 years of medical practice, ranging from 11 to 34 years. Among the informaticists, 58% (7/12) were men, and 92% (11/12) white. All were physicians, except for 2 nurses. On average, patients were 54 years old, 60% (21/35) were women, and 24% (8/35) were reviewing information regarding breast cancer. A majority (25/35, 71%) underwent initial tests or was diagnosed at least 6 months before being contacted for this study. The status of cancer in patients included 43% (15/35) with metastatic cancer and 34% (11/35) with stage 2 cancer or further progressions. Compared with participants enrolled in the study, those who refused participation were slightly younger and mostly men. A full demographic summary is provided in Table 2.

### Themes

We identified two primary themes (1) implementation practices influence attitudes, which describes how involvement, or lack thereof, during the decision-making and execution process of employing portals can impact the sentiment of the oncologists toward them and (2) perceptions of portals as communication tools varies by user type. This theme describes the lens of each stakeholder about how the patient portal is used to transmit and receive information, and contains several subthemes. Textboxes 1 and 2 provide a summary of the themes, subthemes, and representative quotes.

#### Theme 1: Implementation Practices Influence Attitudes

Opinions of oncologists about the portal were shaped by their lack of inclusion and consultation before the portal’s implementation. Unable to voice their concerns about the potential of patients experiencing anxiety by viewing reports on their own, oncologists at the institution, where the study took place, were hesitant to embrace the portal. The portal’s sudden implementation came as a surprise, as stated by the member of the medical informatics committee recalled about the decision to implement portals:

It was pretty uniform amongst all of us [on the committee] that we should...adopt [open access]...and there was not even a ruffle of any discussion about it. We just sort of sneaked it in on people.

Member of Informatics Committee

During the initial rollout, sensitive tests such as pregnancy, HIV, scans and pathology were not visible to patients. However, the more medical informaticists used the system for less sensitive information, the more they believed that transparency was positively transforming patient engagement. After including test results such as scans and pathology, an embargo period of 14 days was established. Shortly thereafter, the embargo was reduced to 4 days. Medical informaticists were aware that physicians in other specialties would be concerned by the shortened embargo, yet they did not receive any resistance nor were concerns voiced after the implementation.

According to oncologists, concerns were not raised because they were largely unaware of the embargo having been reduced to 4 days. A medical informaticist at the location of this study acknowledged this and said:

I don’t think [oncologists] know about it [the reduction in embargo to 4 days].

Indeed, oncologists had limited knowledge of the patient portal’s functionality which is exemplified below,

Provider training [is necessary]. I’d like to know like what patients can see and what the timeline is.

Radiation Oncologist

Among external health systems, where medical informaticists sought buy-in from oncologists, better acceptability was reported. For example, a CMIO in the Western USA first acquired the endorsement of oncologists:

I worked with the chair and we went through all the different reports...He then went back to his group, explained it to the group. The group felt supportive of it as well and we moved on.

CMIO, Western United States

However, the decision-making processes were unique to each institution. Some hospitals in the Western and Eastern United States did not make pathology reports accessible via the portal; a Midwestern hospital authorized a 7-day moratorium on scan reports; and a hospital in the Eastern United States unilaterally decided to implement based on the instructions of the CMIO. 

**Table 1:** Recruitment summary and organization.

Participants			n (%)
**Oncologists (N=46)**			
	**Randomly selected to participate**		22 (48)
		Hematology	11 (24)
		Radiation	10 (22)
		Surgical	1 (2)
	**Enrolled in study**		13 (59)
		Hematology	8 (61)
		Radiation	4 (31)
		Surgical	1 (8)
**Informaticists (N=13)**			
	**Randomly selected to participate**		13 (100)
		Internal	7 (54)
		External	6 (46)
	**Enrolled in study**		12 (92)
		Internal	6 (50)
		External	6 (50)
**Patients (N=78)**			
	**Randomly selected to participate**		72 (92)
		Hematology	45 (63)
		Radiation	23 (32)
		Surgical	4 (5)
	**Referred by physician**		6 (8)
		Hematology	6 (100)
		Radiation	0 (0)
		Surgical	0 (0)
	**Enrolled in study**		35 (45)
		Hematology	24 (69)
		Radiation	8 (23)
		Surgical	3 (9)

**Table 2:** Sociodemographic characteristics of patients (N=35).

Characteristics of the patients		Values
**Sex, n (%)**		
	Female	21 (60)
	Male	14 (40)
Age in years, mean (SD)		53.7 (10.8)
**Race, n (%)**			
	White	21 (60)
	Black	14 (40)
	Asian	0 (0)
**Household income in US $, n (%)**		
	Under $19K	7 (20)
	$20K-$39K	4 (11)
	$40K-$49K	1 (3)
	$50K-$74K	8 (23)
	$75K-$99K	4 (11)
	$100K+	4 (11)
	Prefer not to say	7 (20)
**Education, n (%)**		
	Some high school	1 (3)
	High school graduate	2 (6)
	Some college	7 (20)
	Associate degree	2 (6)
	Bachelor’s degree	13 (37)
	Master’s degree	7 (20)
	Professional degree	2 (6)
	Doctorate	1 (3)
**Area of test/diagnosis^a^, n (%)**		
	Breast	9 (24)
	Hematologic	6 (16)
	Gastrointestinal	5 (13)
	Genitourinary	4 (10)
	Lung	3 (8)
	Sarcoma	3 (8)
	Skin	3 (8)
	Gynecologic	2 (5)
	Other	3 (8)
**Cancer status, n (%)**		
	Metastasized	15 (43)
	Stage 2	4 (33)
	Stage 3	4 (33)
	Stage 4	4 (33)

^a^Each diagnosis/condition counted separately for patients with multiple diagnoses/conditions.

**Representative quotes of theme 1.**

Implementation practices influence attitudes—Responses to question about unawareness of oncologists to the test release timing results:

- *It’s confusing because we brought it down from 2 weeks to 4 days and at the same time we brought the test results down from 72 hours to zero. And it’s hard to keep up with all of that for them. So that’s probably why.* [Informaticist]
- *It’s very confusing because I feel like the health care system just threw it out there and it’s almost like they threw providers and nurses under the bus because you don’t know what the patient is seeing and when and you’re just like, okay.* [Medical Oncologist]

**Representative quotes of theme 2 and its subthemes.**

Perceptions of portals as communication tools varies by user type

- Lack of acknowledgment of the culture and communication processes surrounding the patient-provider relationship in different medical fields
  - *So I know my side in primary pediatrics, I don’t know if I have a full understanding of what oncologists is, their side of it. I think it’s two different, it’s medicine, it’s still sensitive subjects, sensitive discussions.* [Informaticist and Pediatrician]
  - *So in primary care, for the most part we’re talking about laboratory results that are of a routine screening nature...which is potentially a motivator to help them do better with their diet, make sure they take their medications, things like that...I think for cancer patients, it’s different...the big concern is finding out they’ve got recurrent disease or progressive disease before the doctor finds out.* [Surgical Oncologist]

- Patient preferences for receiving information
  - *I do everything digitally so I love just being able to just pop on there and see immediate results and also gave me a history of tracking so if I wanted to be able to look back at something it was easy to do that and then also to communicate with the doctor and whenever I had questions I would post an email for him.* [Patient, Lymphoma]
  - *It is helpful to be able to go on and check it out. I had a CT scan, I know I can go on there in just a couple days and check it out and see what the radiologist wrote. And then I find that very comforting.* [Patient, Lung Cancer]
  - *The best way would be to go to the doctor direct about it...I think finding out from the doctor is the best way. Obviously.* [Patient, Stomach Cancer]

- Type of information disclosed
  - *I just got back from the doctor yesterday and I had to wait 2 or 3 weeks to find out the results of my CAT scan. Because they had thought that it might have been lung cancer. So I’ve been worried...[Using the portal] would have been very helpful.* [Patient, Sarcoma]
  - *There is established literature that says that the patient, physician discussion of breaking bad news, is an important role of a physician and that it’s done compassionately in person, much better than on your own and over the phone.* [Medical Oncologist]
  - *I do not want to read on an MRI that my diagnosis is cancer. I would rather have a doctor discuss that with me before I have to review it online.* [Patient, Sarcoma]
  - *I had very difficult interactions in the past trying to break news over the phone for somebody who didn’t want to wait for their appointment because they were expecting one outcome and they saw another. And there’s no further counseling that can take place. They’re in the middle of their own workplace environment. They don’t have their family’s support there, they’re not braced for these types of things and it was a very negative experience.* [Medical Oncologist]

Uncertainty about patient access was shared by medical informaticists at other institutions, but the benefits of transparency outweighed concern. Benefits included greater patient engagement and patient vigilance. For instance, a CMIO lauded the capability of patients to easily share information with family members and being able to discover inconsistencies in their record. Speed was also important, as quoted below:

Patients are really eager to be able to have both rapid access and more complete access.

Medical Informaticist, Western United States

#### Theme 2: Perceptions on Portals as Communication Tools Vary by User Type

Oncologists, medical informaticists, and patients–the three stakeholders–cited examples of portal usage and how the portal was incorporated into their daily lives. The following subthemes emerged: (1) lack of acknowledgment of the culture and communication processes surrounding the patient-care-provider relationship in different medical fields, in which oncologists and medical informaticists explained the norms of communicating with patients in their fields and differences in the meaning of paternalism; (2) patient preferences for receiving information, including whether patients view the portal before communicating with their oncologist and the ideal setting to receive diagnostic results, and (3) type of information disclosed, wherein the patient’s phase of diagnosis determined their comfort level while using the portal.

##### Subtheme 2.1: Different Culture and Communication Processes

A cultural divide was present between oncologists and medical informaticists: none of the informaticists involved in this study had a background in oncology. Most medical informaticists specialized in internal or family medicine, but they recognized that viewing information about potential metastasis could be different from primary care issues. The apprehension of oncologists toward the patients accessing medical information via patient portals stemmed from their belief that tests for cancer were more sensitive than common laboratory results screened during primary care visits. However, medical informaticists downplayed potential risks by citing existing literature indicating that it was not a problem and the fact that they have not personally encountered such negative incidents. Ultimately, the purview of informaticists was championed by a CMIO and internist, who recommended:

[Oncologists must] Get out of their comfort zone and recognize this as a new era.

However, oncologists remained steadfast in their belief that the patient-provider relationship, as well as the utilization of patient portals would be unique in cases of cancer and differed from primary care.

The stakes in oncology are really high. In primary care, if you get an x-ray of someone’s shoulder, you’re looking for arthritis. If I get an x-ray...I’m looking for a bone metastasis.

Radiation Oncologist

Due to the precariousness of cancer, oncologists purposefully scheduled face-to-face meetings. However, interviews revealed that the patient portal has increasingly driven oncologists to communicate over the phone. A medical oncologist recalled the importance of face-to-face interactions:

There are times when you need to be able to hold hands...You need to be able to see them...to help them understand what that news really means.

Medical Oncologist

Despite the beliefs of the oncologists that in-person discussions were necessary, informaticists considered any delay in disclosing results as paternalism. A family physician and informaticist decried the process of patients returning to the hospital to learn diagnoses and wondered:

If this was my MRI, would I want to wait for 2 weeks? Hell no...If that’s right for me, why isn’t it right for my patients?

Family Physician

Oncologists, who were concerned about patients viewing scans or pathology results on their own using the portal, suggested alternative solutions such as permitting a function that allows tests to be released after a physician views it, but that type of functionality was not technologically feasible at the time when this study was conducted. Medical informaticists did not have the technology modify the visibility of certain tests for certain departments.

##### Subtheme 2.2: Patient Preferences for Receiving Information

Oncologists and medical informaticists were ardent in their respective beliefs, but these beliefs were somewhat disconnected to the perceptions of patients on the role of portals. Patients being treated or screened for cancer displayed attitudes and behaviors suggesting that despite some hesitation, they were largely comfortable using the portal. More than half (19/35, 54%) of patients interviewed retrieved test results or scan reports using the portal before speaking with a provider. None of the patients expressed shock or extreme distress. In fact, a patient with a rare blood cancer appreciated the ability to discover the diagnosis on her own and said:

I learned about this in the privacy of my home where it’s quiet.

The lack of distressed patients was in accordance with the limited number of negative incidents as cited by oncologists. Most (7/13, 54%) oncologists did not experience a negative incident, but instead mentioned anticipated dangers or negative experiences of their colleagues. Among the (6/13, 46%) oncologists who cited specific instances, each described a patient who suffered anxiety believing that their cancer had reoccurred. Due to such cited examples, 3 patients participating in the study were referred. When asked to describe their incident, a woman said that she experienced *“no stress,”* while a breast cancer patient said that her anxiety levels were raised *“a little bit.”* In general, cancer patient responses to portal usage were influenced by their stated preferences for communication with their oncologist and the types of information being disclosed.

Although patients preferred swift results, most patients believed that in-person meetings or phone calls were ideal to receive a diagnosis. A woman with breast cancer remembered her consultation and said:

By meeting with her [the oncologist], it made it more personal that she actually cared about the outcome versus, the report will be up there, you can read if you have cancer.

Breast Cancer Patient

However, the portal was a welcome alternative if either a phone call or in-person meeting caused any delay in receiving results. Another breast cancer patient said:

I would’ve liked to have had the oncologist call me and say it’s not cancer or, just make the report available on the portal. Rather than having it held before I could view it...I would’ve liked to have had it as soon as it was available.

Breast Cancer Patient

While oncologists were fearful that patients would experience distress, most patients appreciated the ability of advanced access to medical reports, because as a breast cancer patient said:

When I am in the doctors’ office, I’m not blindsided by information...there’s no surprises.

Armed with their medical information in advance, patients claimed that face-to-face appointments were more productive with oncologists. Oncologists agreed that advanced access can improve engagement during consultations, as well as assist patients after appointments by allowing them to review information that was discussed.

##### Subtheme 2.3: Types of Information Disclosed

Patients with a previous cancer diagnosis or in the survivorship phase spoke positively about using the patient portal during or after treatment. However, they expressed reservations about the prospect of learning a cancer diagnosis through the patient portal. A lung cancer patient imagined the difficulties in not being able to get immediate answers to her questions, while a breast cancer patient stated:

I guess if I had found out over the Internet instead of face-to-face with a doctor that I had cancer the first time, it might be a little daunting.

Breast Cancer Patient

Patients also placed different values on different types of tests. A man with sarcoma said:

I don’t mind reading my blood levels, but if we’re talking about...worsening or getting better, those things should come from the physician.

Sarcoma Patient

A surgical oncologist agreed that scans, pathology, and biopsy reports should be disclosed by the physician. He went on to say,

[Reports] require a fair amount of explanation, particularly to a layperson who doesn’t understand them.

Physician

Relatedly, a medical oncologist worried that a rift may form between the patient and oncologist because positive findings may be present on the report, even though they are insignificant. He wondered if patients would trust oncologists less because some patients may be skeptical of what their oncologist was telling them, after reading the report themselves.

## Discussion

### Principal Findings

Health care delivery increasingly relies on technology to manage aspects of patient care [37]. In oncology, technology (eg, patient portals) is still novel, but its introduction assures implications for both patients and clinicians. In our interviews about the perceptions and use of the patient portal in oncology, we discovered divergent views and no clear blueprint for properly implementing such a system. The introduction of electronic records and health information technology in general has been known to profoundly affect health systems, impacting health care delivery [38] and altering relationships among patient care providers [39]. We found that while informaticists advocated for full transparency, oncologists preferred more control over the delivery of information, even though their fears of patient distress were generally unrealized. One explanation for this divide could be that none of the informaticists specialized in oncology and were mainly primary care physicians. Although it is not atypical for primary care physicians to break bad news, oncologists frequently deal with high mortality rates and face highly stressful situations on a daily basis, commonly addressing topics such as death, dying, and palliative care [40].

When faced with complex, potentially life-threatening, medical information through the portal, patients in our study seldom expressed concern or felt that they experienced additional distress. In fact, some patients found solace in being able to review their results on their own terms. This is consistent with limited studies that have measured anxiety among cancer patients who accessed test results through patient portals and also experienced low levels of distress [41-44].

Patient’s lack of concern may be explained by the fact that the majority of participants (25/35, 71%) had progressed more than 6 months from their initial diagnosis and 43% (15/35) had developed metastasized cancers. These patients had been managing their disease with treatment over the course of several months or years, may have gained knowledge and experience, and thus may have become desensitized to viewing their medical information compared with the patients confronted with an initial diagnosis.

Our findings that the majority of patients who were interviewed reviewed test results or scan reports before speaking with a provider is noteworthy. Perhaps, during an initial oncology consultation, oncologists should note that potentially threatening risk information can be available by using the portal and identify whether the portal is the patient’s preferred communication channel. Oncologists should also recognize that their own preferred method of delivering bad news via in-person disclosures [45,46] accompanied by emotional support, may need to be modified in the light of patient preferences for immediate delivery of results [47], even when they are abnormal [48]. Similar to previous studies examining use of the Internet by patients to manage their cancer care, computer-savvy patients may necessitate the need for providers to modify the way they interact with patients [49].

### Limitations and Future Directions

Despite the attempt to broaden the sample with representatives from other health systems, our results may still not extend beyond the health system in which this study was conducted. Similarly, the sample may include a proportal bias, since only patients enrolled in the portal were eligible, and almost all informaticists who advocated for portals agreed to participate. In addition, the average age of our sample was 54 years. It is possible that inclusion of younger patients would produce additional perspectives. Moreover, we did not recruit patients with newly diagnosed disease or new evidence of metastatic disease. Although it was important to report differences of recollections between perceptions of oncologists and patients, all patients in the study had received their diagnosis before the study, which highlights the need for further research to examine real-time responses using larger samples instead of recollected responses. In addition, to further illuminate our knowledge, future work is warranted to explore the attitudes and perceptions of patients with a broader range of disease sites and stages and to include patients who are early in their cancer trajectory. We also plan to involve patient’s family members and caregivers, who are often avid patient portal users [50]. Further research could also focus on measuring how other highly specialized medical departments use patient portals and whether training programs and targeted education about portal use is an effective way of ensuring that portals are being used to optimize the quality of patient care.

### Conclusions

Our findings indicate that the complexity of communicating medical information related to oncology varies the utility of patient portals. Although most patients prefer in-person consultations to learn about their condition, the patient portal is rapidly being accepted and may force oncologists to alter their communication habits.

Most cancer patients who participated in the study checked their laboratory results or scan reports in the portal before being contacted by their provider. Although most were relatively comfortable with this manner of disclosure, few patients were checking an initial diagnosis, wherein the preferred disclosure method was phone or face-to-face. As informaticists and other high-ranking personnel within health systems make tests available to patients through the portal, it is necessary that in-depth discussions with specialized areas of medicine, such as oncology, must take place. The implementation process across the entire health system is unlikely to succeed if certain groups are not able to give their input about critical features of the portal. However, oncologists should understand that the delivery of medical information via patient portals is inevitable, and therefore, they must take efforts to discuss the portal with patients. Although using the patient portal as a new channel to transmit medical information will require oncologists to alter their communication methods with patients in the short term, establishing best practices will allow oncologists to incorporate new techniques before portal adoption.

In summary, we sought the perspectives of patients, oncologists, and informaticists to understand the advantages and disadvantages of patient portals in the oncology setting. Results indicate that the portal may provide benefits, such as enabling more productive in-person appointments. However, education and training is necessary to inform patients and oncologists of the portal’s advantages. We anticipate that this study helps generate additional insights that will help future research in using patient portal technology in oncology effectively.

## Abbreviations

CMIO: chief medical information officer
CIT: critical incident technique
